# A Mutational Analysis of Residues in Cholera Toxin A1 Necessary for Interaction with Its Substrate, the Stimulatory G Protein Gsα

**DOI:** 10.3390/toxins7030919

**Published:** 2015-03-18

**Authors:** Michael G. Jobling, Lisa F. Gotow, Zhijie Yang, Randall K. Holmes

**Affiliations:** 1 Department of Immunology and Microbiology, School of Medicine, University of Colorado Denver, 12800 E 19th Ave, Aurora, CO 80045, USA; E-Mail: michael.jobling@ucdenver.edu; 2 Department of Biology, Metropolitan State University of Denver, P.O. Box 173362, CB 53, Denver, CO 80217, USA; E-Mail: lgotow@msudenver.edu; 3 Atila Biosystems Inc., 740 Sierra Vista Ave, Unit E, Mountain View, CA 94043, USA; E-Mail: zhijie.yang@atilabiosystems.com

**Keywords:** cholera toxin, Gs alpha, ADP-ribosylation

## Abstract

Pathogenesis of cholera diarrhea requires cholera toxin (CT)-mediated adenosine diphosphate (ADP)-ribosylation of stimulatory G protein (Gsα) in enterocytes. CT is an AB5 toxin with an inactive CTA1 domain linked via CTA2 to a pentameric receptor-binding B subunit. Allosterically activated CTA1 fragment in complex with NAD^+^ and GTP-bound ADP-ribosylation factor 6 (ARF6-GTP) differs conformationally from the CTA1 domain in holotoxin. A surface-exposed knob and a short α-helix (formed, respectively, by rearranging “active-site” and “activation” loops in inactive CTA1) and an ADP ribosylating turn-turn (ARTT) motif, all located near the CTA1 catalytic site, were evaluated for possible roles in recognizing Gsα. CT variants with one, two or three alanine substitutions at surface-exposed residues within these CTA1 motifs were tested for assembly into holotoxin and ADP-ribosylating activity against Gsα and diethylamino-(benzylidineamino)-guanidine (DEABAG), a small substrate predicted to fit into the CTA1 active site). Variants with single alanine substitutions at H55, R67, L71, S78, or D109 had nearly wild-type activity with DEABAG but significantly decreased activity with Gsα, suggesting that the corresponding residues in native CTA1 participate in recognizing Gsα. As several variants with multiple substitutions at these positions retained partial activity against Gsα, other residues in CTA1 likely also participate in recognizing Gsα.

## 1. Introduction

Cholera toxin (CT) is a major virulence factor produced by *Vibrio cholerae*, the causative agent of human cholera [1]. The action of CT on intestinal epithelial cells results in net fluid secretion and the massive watery diarrhea characteristic of cholera. CT is the prototype AB5 toxin, consisting of a single enzymatic A subunit non-covalently linked to a homopentameric B subunit [2]. The A and B polypeptides are made as pre-proteins, secreted into the periplasm, processed to mature forms, and assembled into catalytically inactive holotoxin that is exported across the outer membrane. CT binds to lipid raft-associated ganglioside GM1 receptors on enterocytes in the small intestine, is internalized by endocytosis, and traffics via the retrograde pathway through the Golgi to the endoplasmic reticulum (ER) [3]. CT is susceptible to proteolytic nicking of its A subunit within a disulfide-linked loop between the CTA1 and CTA2 domains, and subsequent reduction of that disulfide bond generates non-covalently-linked CTA1 and CTA2 fragments. Nicking can be accomplished either by *Vibrio*-produced proteases or proteases of enterocytes to which CT is exposed during uptake and internalization. Reduction occurs in the ER of intoxicated enterocytes, after which chaperone-facilitated dissociation from holotoxin initiates retrotranslocation of reduced CTA1 into the cytosol. Although the reduced CTA1 fragment has a basal level of catalytic activity, it is allosterically activated in the cytosol by binding to the GTP-bound forms of cellular co-factors called ADP-ribosylation factors (ARFs) [4,5]. Allosterically-activated CTA1 ADP-ribosylates and activates the alpha subunit of the stimulatory heterotrimeric G-protein (Gsα) resulting sequentially in constitutive activation of adenylate cyclase, increased production of cAMP, activation of protein kinase A, phosphorylation and activation of the cystic fibrosis transmembrane conductance regulator chloride channel, and enhanced secretion of Cl^−^ ions into the intestinal lumen [6]. These events, plus, cAMP-dependent inhibition of Na^+^-uptake [7], cause fluid loss into the intestine that presents clinically as watery diarrhea.

CTA1 undergoes several conformational changes during its transition from the catalytically latent CTA1 domain in holotoxin to the nicked, reduced and ARF-bound active CTA1 fragment that is presumed to ADP ribosylate Gsα. The structure of a nicked but not reduced form of the related heat-labile enterotoxin LT-I showed no major conformational differences from un-nicked toxin [8]. The latent catalytic site within the CTA1 domain of holotoxin is occluded by an “active site” loop (consisting of residues 47–56) that is held in place by interactions with an “activation loop” (consisting of residues 25–36) that also interacts with CTA2 [9]. The structure of an enzymatically inactive R7K variant of LT provided early evidence that the corresponding activation loop of LT was disordered. Analysis of that structure generated a model whereby nicking and reduction of the holotoxin were proposed to initiate activation of the corresponding A1 domain by a three-step process, starting at the site of nicking and reduction, propagating to the active site, and involving: (1) increased flexibility of the long alpha helical segment of the A2 domain leading to disruption of its interaction with the “activation loop”; (2) increased flexibility of the “activation loop” leading to disruption of its interaction with the “active site” loop; and (3) increased flexibility of the “active site” loop enabling it be displaced and permit entry of the substrates NAD and the target arginine residue of Gsα into the active site [10]. Additional support for this model was provided by crystal structures of a CT variant with a Y30S substitution in the “activation” loop that permitted the holotoxin to exhibit intrinsic enzymatic activity without any requirement for proteolysis or reduction [9]. The “activation” loop in each of several crystal forms of this Y30S holotoxin variant was disordered, and the “active site” loop displayed varying degrees of order.

**Figure 1:**
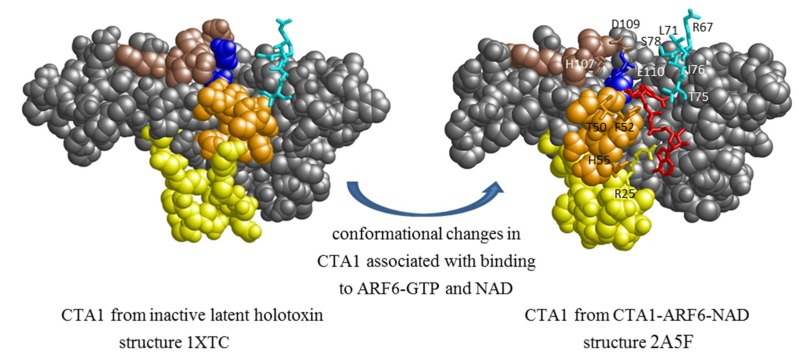
Conformational changes in cholera toxin A1 subunit CTA1 associated with binding to ADP-ribosylation factor 6 (ARF6)-GTP and NAD and predictions of candidate residues in CTA1 that may participate in Gsα binding. Space-filling projection of CTA1_wt_ from the crystal structures of latent holotoxin (1XTC, left) or CTA1_E110D,E112D_ with NAD^+^ and ARF6 (2A5F, right). Coloring and feature annotation is based on Figure 4B in reference [12]. ARF6 (not shown) binds to an interface on the rear of this projection. Residues 25–40 (activation loop) are yellow, residues 47–56 (active site loop, including knob in ARF6-bound form) are orange, residues 104–110 (ARTT motif) are brown, and active site residues 110 (stick) and 112 (space-fill) are blue. NAD is shown as sticks and is red. Each residue substituted by an alanine in this study is numbered, shown in stick format, colored cyan or as described above, and identified by the standard IUPAC one-letter code and position.

CTA1 has low intrinsic enzymatic activity *in vitro*, but interaction of CTA1 with any of several eukaryotic ARFs results in allosteric activation of its catalytic activity. A bacterial two-hybrid analysis of CTA1 and ARF6 showed that they form a tight interaction complex and identified residues in CTA1 that are required for binding to ARF6, thereby identifying a potential interaction interface [11]. Subsequent studies led to production in *E. coli* and purification of a complex containing a catalytically inactive CTA1 variant (with E110D and E112D substitutions) and ARF6-GTP, and comparisons of crystal structures of this complex (with or without bound NAD) [12] showed that residues 25–33 within the “activation loop” (residues 25–40) of CTA1 rearrange from an ordered coil in the catalytically latent holotoxin to a short amphipathic helix in the CTA1:ARF6-GTP complex (see Figure 1, Results and Discussion). Additional conformational changes that occur in CTA1 when it binds to ARF6-GTP include re-arranging residues 48–52 within the “active site” loop (residues 47–56) to form a knob near the active site and positioning the ADP ribosylating turn-turn (ARTT) motif (residues 104–110) near the active site. The ARTT motif is conserved among several ADP-ribosylating toxins (including pertussis toxin, CT, LT, diphtheria toxin, and *Pseudomonas aeruginosa* exotoxin A), and it participates in target protein recognition in some of them [13,14]. Taken together, these findings allowed us to predict several surface-exposed residues near the active site of CTA1 that might participate in binding and recognition of Gsα as an ADP ribosylation target. Nevertheless, Gsα alone is a poor substrate for CT, and under certain conditions CT can also ADP-ribosylate other G proteins including Giα [15,16,17]. Current biochemical and cell biological evidence suggests that the preferred *in vivo* substrate for CT may be a short-lived heterotrimeric Gαβγ complex bound to an activated G protein coupled-receptor (GPCR) and with Gsα in a nucleotide-free state—*i.e*., after release of bound GDP but before Gβγ dissociates from Gsα and before Gsα binds GTP [18].

*In vitro*, CT can also ADP-ribosylate several small guanidino-group-containing artificial substrates that include agmatine [19] and diethylamino-(benzylidineamino)-guanidine (DEABAG) [20]. Structural and mutational studies identified several critically important residues of CTA1 or the related LTA1 that are required for enzymatic activity and NAD-substrate/ARF-co-factor binding [11,21,22,23], but the molecular interactions of CTA1 with Gsα are not well characterized. We hypothesized that ADP-ribosylation of a small artificial substrate like DEABAG would depend primarily on its interactions with ligands within the CTA1 active site, whereas ADP-ribosylation of the much larger protein substrate Gsα would likely require additional interactions with CTA1 ligands outside the active site. In this study, we introduced alanine substitutions into CT holotoxin at selected surface-exposed positions near the CTA1 active site, screened the purified, nicked and reduced holotoxin variants *in vitro* to identify ones that retained full or nearly-full ability to ADP ribosylate DEABAG but exhibited decreased ability to ADP ribosylate Gsα, and thereby identified specific amino acid residues in CTA1 that likely participate in recognition and binding interactions with Gsα.

## 2. Results and Discussion

Figure 1 compares the conformation of a catalytically inactive CTA1_E110D,E112D_ variant in complex with ARF6-GTP and NAD with the proenzyme form of wild-type CTA1 in native (un-nicked and un-reduced) CT. Based on the novel conformation of the allosterically-activated CTA1_E110D,E112D_ fragment in complex with ARF6-GTP and NAD, we selected several surface-exposed residues near the active-site cleft to investigate as possible contributors to the interaction interface between CTA1 and Gsα. The selected residues (see Figure 1) are as follows: R25 at the *N*-terminal end of the activation loop (residues 25–40); T50 and F52 (within the knob formed upon activation) plus H55 in the active-site loop (residues 47–56); R67 (not in a defined motif); L71, T75, I76, and S78 in a loop (residues 71–78) that may be comparable to the PN loop in the Ia fragment of the ADP-ribosylating iota toxin from Clostridium perfringens [24,25] (although lacking any amino-acid homology); and H107, D109, and E110 in the ARTT motif. It is noteworthy that the positions of most of these selected residues differ significantly between the proenzyme and allosterically-activated conformations of CTA1. Mutations encoding single or multiple alanine substitutions for these selected residues were introduced by site-directed mutagenesis into the *ctxA1*-coding region of the inducible holotoxin-encoding plasmid clone pARCT5. Introduced mutations were confirmed by restriction digests of PCR amplicons of the *ctxA1* coding region to detect the novel restriction site(s) introduced with the alanine codon(s), followed by DNA sequencing to confirm that no unintended mutations were introduced in the cloned genes.

**Figure 2:**
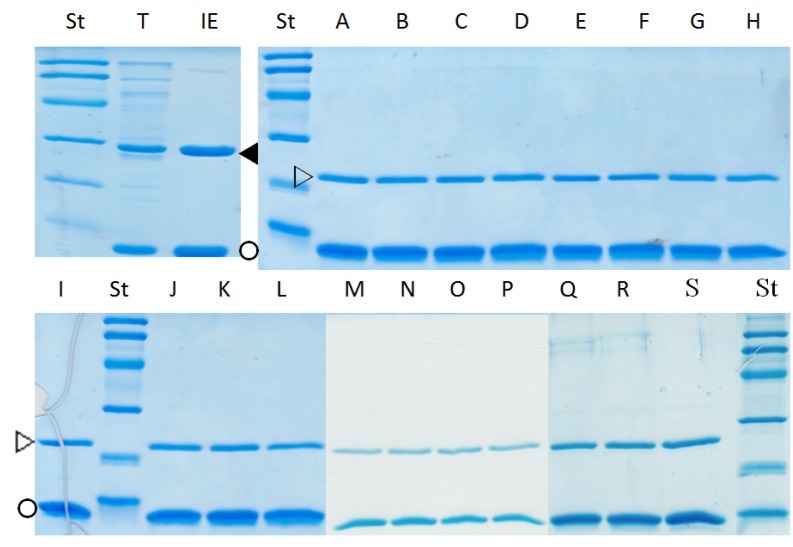
Purification and trypsin resistance of wt CT and CT variants with alanine substitutions in CTA1.SDS-PAGE analysis (15% gels) of wt and variant holotoxins. All samples were boiled with 5% β-ME prior to loading and electrophoresis at 200 V, 45 mins, followed by colloidal staining with Coomassie Blue [26]. Top Left panel: St, protein standards; T, partially purified wt CT after Talon purification; IE, purified wt CT after ion-exchange chromatography. Lanes T and IE each contain 2.5 µg of un-nicked toxin. Top Right panel: analysis of 2.5 µg samples of trypsin-treated wt and single- or double-alanine-substitution CT variants. St, protein standards; A, wt CT; B, CT-H55A; C, CT-R67A; D, CT-L71A; E, CT-S78A; F, CT-D109A; G, CT-H55A/L71A; H, CT-H55A/S78A. Bottom panel, analysis of 2.5 µg samples of trypsin-treated double- or triple-alanine-substitution CT variants. I, CT-H55A/D109A; St, protein standards; J, CT-L71A/D109A; K, CT-S78A/D109A; L, CT-L71A/S78A; M, CT-R67A/S78A; N, CT-R67A/D109A; O, CT-H55A/L71A/S78A; P, CT-H55A/L71A/D109A; Q, CT-H55A/R67A; R, CT-H55A/R67A/L71A; S, CT-R67A/L71A; St, protein standards. Molecular masses: protein standards (top to bottom), 97, 66, 45, 31, 21.5 and 14 kDa; un-nicked CTA, 28 kDa (filled triangle); CTA1, 21.5 kDa (open triangle); CTB, 11.5 kDa (circle); CTA2, 6.5 kDa (does not stain). The figure is a composite of multiple gels that differ only in the degree of destaining.

Wild type (wt) and variant forms of CT holotoxin (listed below in the legend for Figure 2) were produced in *E. coli*, isolated from cellular extracts by Talon affinity chromatography, and further purified by ion-exchange chromatography. Holotoxins produced in *E. coli* have an intact CTA polypeptide that must be cleaved within the disulfide-linked loop joining the CTA1 and CTA2 domains and also reduced to produce the catalytically active CTA1 fragment. One sensitive measure of correct folding and assembly of variant toxins is their resistance to limited trypsin digestion. The CTA subunit of native toxin is nicked to give stable CTA1 and CTA2 fragments, while variants that are not folded correctly have A subunits that are rapidly and progressively degraded in the presence of limiting amounts of trypsin [22]. Therefore, we assessed the effects of limited trypsin digestion on the wt and variant forms of CT constructed for this study to see if any of the introduced alanine substitutions altered toxin stability. Each CT variant behaved comparably to wt CT and was nicked by trypsin to produce a stable A1 fragment, showing that these alanine substitutions did not measurably affect toxin assembly or resistance to limited trypsin digestion (Figure 2).

**Figure 3:**
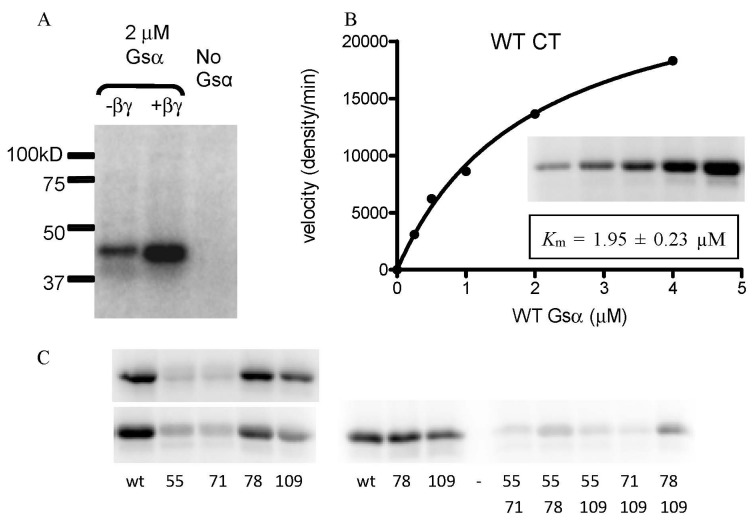
*In vitro* ADP-ribosylation of Gsα by nicked and reduced wt and variant forms of CT in the presence of ARF6-GTP and Gβγ. Top left panel (**A**), Gβγ is required for efficient ADP-ribosylation of Gsα by wt CT. Top right panel (**B**), determination of apparent *K*_m_ for ADP-ribosylation of Gsα by wt CT. Lower left panel (**C**), reproducibility of Gsα ADP-ribosylation assay. Independent assays (upper and lower sections) were performed one week apart with (L to R) wt CT, CT-H55A, CT-L71A, CT-S78A, and CT-D109A. Lower right panel, ADP-ribosylation of Gsα by representative CT variants: (L to R) wt CT, CT-S78A, CT-D109A, empty lane, CT-H55A/L71A, CT-H55A/S78A, CT-H55A/D109A, CT-L71A/D109A, and CT-S78A/D109A.

To analyze the biochemical and biological activities of these wt and variant CT holotoxins, we tested them in three assays: (1) ADP ribosyltransferase activity *in vitro* with the native substrate Gsαβγ; (2) ADP ribosyltransferase activity *in vitro* with the artificial substrate DEABAG; and (3) toxicity for cultured Y1 adrenal cells (exhibited by increased intracellular cAMP). ADP-ribosylation of Gsα by CT was facilitated by the addition of Gβγ (Figure 3A), and Gβγ was included in all subsequent assays for ADP ribosylation of Gsα. The apparent *K*_m_ for Gsα during ADP-ribosylation by wt CT was 1.95 ± 0.23 µM (Figure 3B). This *K*_m_ for Gsα is close to the calculated (low µM) concentration of Gsα in rat myocytes (based on approximately 5 × 10^7^ molecules of both short and long Gsα isoforms per cell [27] and an average cell volume of 34 pL [28]). We then determined the activity of each of our CT variants for ADP-ribosylation of Gsα under identical conditions, and representative results are shown in Figure 3C.

**Figure 4:**
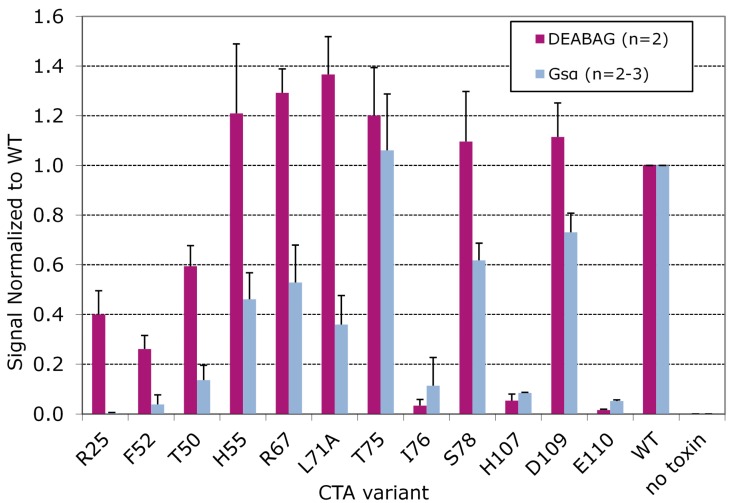
Relative activity of CTA variants in ADP-ribosylation assays with diethylamino-(benzylidineamino)-guanidine (DEABAG) or Gsα.

Quantitative ADP ribosylation assays catalyzed by wt CT and each of the CT variants were performed separately with Gsα and DEABAG as acceptor molecules, and for each set of assays the activity obtained with wt CT was set to 1.0 and the activity of each CT variant was normalized to the activity of wt CT (Figure 4). The CT-T75A variant did not differ significantly in activity from wt CT and was not considered further. The CT-I76A, CT-H107A and CT-E110A variants exhibited greatly decreased ADP-ribosyltransferase activity against both DEABAG and Gsα.

Because the alanine substitutions in these three CT variants nearly abolished catalytic activity, we could not evaluate whether or not the I76, H107 and E110 residues contributed to recognition of Gsα by CTA1. The CT-R25A, CT-F52A, and CT-T50A variants exhibited partial activity against DEABAG but much less activity against Gsα, suggesting that the wt residues at these positions contribute both to intrinsic catalytic activity of CTA1 and to recognition of Gsα by CTA1. Finally, the CT-H55A, CT-R67A, CT-L71A, CT-S78A, and CT-D109A variants exhibited full activity against DEABAG but substantially decreased activity against Gsα, suggesting that the alanine substitutions at each of these positions decreased the ability of CTA1 to recognize Gsα without affecting the intrinsic catalytic activity of CTA1.

**Figure 5:**
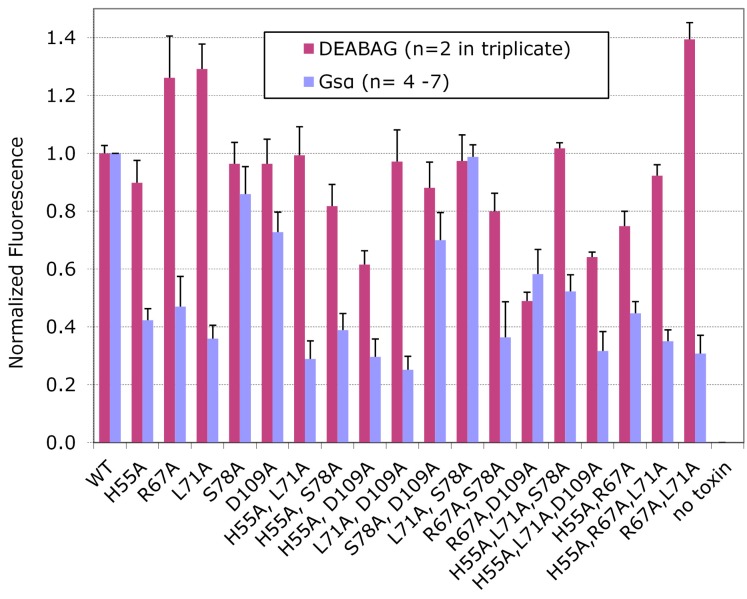
Effects of single-, double-, and triple-alanine-substitutions on the ratios of enzymatic activity for Gsα and DEABAG, normalized to the activities of wt CT.

We also generated CT variants with two or three alanine substitutions distributed among positions 55, 67, 71, 78, or 109 in CTA1 to assess their cumulative effects on holotoxin assembly, susceptibility to degradation by trypsin, and catalytic activity against DEABAG and Gsα (Figure 5).

We confirmed that these multiple substitutions did not affect toxin assembly or susceptibility to degradation by trypsin (see Figure 2, lanes G-I, L-N, and Q for representative data on double mutants; and lanes O-P and R-S for representative data on triple mutants). All of these doubly- and triply-alanine-substituted CT variants retained full or partial catalytic activity with DEABAG (Figure 5), and only the CT-H55A/D109A, R67A/D109A, and CT-H55A/L71A/D109A variants exhibited less than 75% of wt CT catalytic activity with DEABAG.

Most of the CT variants with multiple alanine substitutions retained ADP-ribosyltransferase for Gsα that was only slightly less than the activity of the least active of the corresponding single-alanine-substitution variants. In contrast, the CT-R67A/L71A double-substitution variant was slightly more active with DEABAG and slightly less active with Gsα than the CT-R67A and CT-L71A single-substitution variants, and it was substantially more active with DEABAG and less active with Gsα than wild-type CT. Furthermore, the CT-L71A/S78A double substitution variant had significantly greater activity with Gsα than the CT-L71A single substitution variant. We speculate that the diethylamino-benzylidine moiety of DEABAG interacts with CTA1 and that the R67A, L71A, or R67A plus L71A substitutions may increase CTA1 activity with DEABAG by modifying that interaction. It is also striking, that the CT variants with multiple alanine substitutions in CTA1 did not show dramatically greater losses of enzymatic activity with Gsα than the corresponding variants with single alanine substitutions.

We determined apparent *K*_m_’s for DEABAG and for Gsα in assays with wt CT and with each of the five CT variants that exhibited preferential loss of catalytic activity with Gsα and retention of full catalytic activity with DEABAG (Table 1). Each of these 5 CT variants had an apparent *K*_m_ for DEABAG that was comparable or slightly lower than that of wt CT. In contrast, the CT-R67A and CT-L71A variants had apparent *K*_m_’s for Gsα that were somewhat higher than that of wt CT. Furthermore, wt CT and the CT-H55A, CT-R67A and CT-L71A variants had comparable *K*_m_’s for NAD (in the presence of 2 mM DEABAG) that were also close to the reported intracellular concentration for NAD (0.37 mM) in mouse erythrocytes [29].

**Table 1:** *K*_m_ determinations for CTA1 variants with Gsα, DEABAG or NAD.

CT Variant	Gsα (µM)	DEABAG (mM)	NAD (mM)
native	1.66 ± 0.88 *	2.9 ± 1.3	0.354
H55A	1.8 ± 0.4	1.8 ± 0.3	0.334
R67A	4.5 ± 1.2	2.4 ± 0.5	0.382
L71A	2.5 ± 0.5	1.7 ± 0.4	0.299
S78A	1.5 ± 0.3	1.5 ± 0.2	ND
D109A	1.0 ± 0.1	3.2 ± 0.5	ND
H55A + L71A	8.6 ± 4.0	ND	ND
H55A + S78A	8.6 ± 4.3	ND	ND
R67A + L71A	5.0 ± 1.0	ND	ND
H55A + R67A + L71A	3.3 ± 0.6	ND	ND

* *n* = 10 (all others are *n* = 2–3; ND, not done).

**Figure 6:**
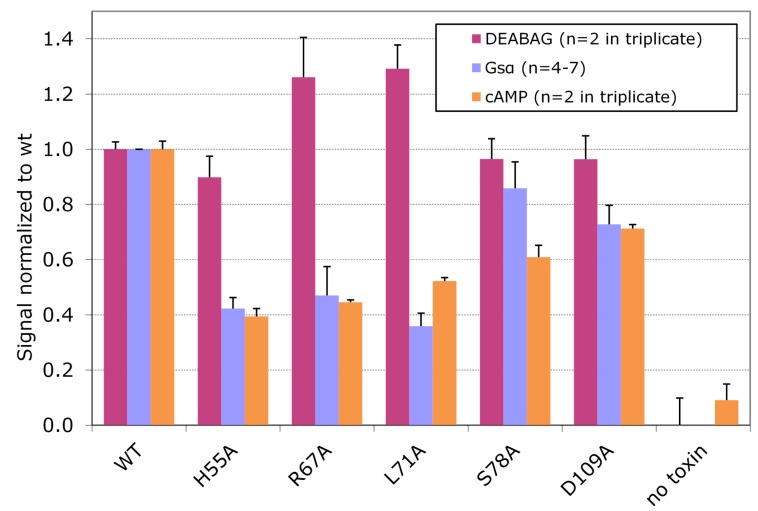
Normalized *in vitro* enzymatic activities with DEABAG or Gsα and normalized intracellular cAMP concentrations in intoxicated Y1 cells for selected CT variants *vs.* wt CT.

The effects of wt CT on cultured Y1 adrenal cells are mediated by increases in the intracellular concentration of cAMP produced as a consequence of intoxication. We performed quantitative assays for intracellular cAMP in Y1 adrenal cells after exposing them to wt CT or to each of the five CT variants associated with full ADP ribosylating activity against DEABAG and selective loss of ADP ribosylating activity against Gsα. Figure 6 compares the values for the *in vitro* ADP-ribosylation activity with DEABAG, the in vitro ADP-ribosylation activity with Gsα, and the intracellular cAMP concentration for each of the CT variants, all normalized to values set to 1.0 for the corresponding results obtained with wt CT. These results show that the *in vivo* cAMP concentrations in Y1 adrenal treated with each of these five CT variants correlate well with the ADP ribosyltransferase activities of the variants against Gsα, but not against DEABAG, and support the conclusion that the residues replaced by alanine substitutions in these variants are directly involved in interactions of CTA1 with Gsα.

Toxin assays were done as described in experimental section, using equal amounts of wt or variant activated holotoxins (2 µg for *in vitro* enzymatic assays and 1 ng for *in vivo* cAMP assay). Calculated activities were then normalized to wt levels (wt CT = 1.0) and plotted for each CT variant, showing SEM for replicates.

In this study, we identified five amino acids (H55, R67, L71, S78, and D109) located near but not in the active site of CTA1 that are likely involved in interactions with the Gsα substrate, based on the demonstration that single alanine substitutions at each of these positions decreased the ADP ribosylating activity of the corresponding CT variants for Gsα but not for the small artificial substrate DEABAG. We also identified three amino acids (R25, T50, and F52) that likely participate in interactions with Gsα substrate but also affect intrinsic catalytic activity, based on the demonstration that single alanine substitutions at each of these positions decreased the ADP ribosylating activity against both substrates, but caused substantially greater loss of activity for Gsα than for DEABAG. Because double and triple alanine substitutions did not cause substantially greater loss of ADP ribosylating activity with Gsα than single alanine substitutions, it seems likely that other residues in CTA1, yet to be identified, also make important contributions to binding interactions between CTA1 and Gsα. If the binding interface between CTA1 and Gsα is large and complex, the consequences of any single alanine substitution for a residue in CTA1 that contributes to that interface may be small. It would be interesting in future studies to determine if more dramatic changes in substrate recognition specificity of CTA1 could be accomplished by substituting residues at the positions identified in the current study that would introduce larger side groups with various properties into the interface (instead of the methyl group provided by an alanine substitution), thereby creating potential steric hindrance as well as altering the local environment within the interface with respect to its hydrophobicity, hydrophilicity or charge. There is only one available structure for an arginine-specific ADP-ribosylating toxin in complex with its substrate, that of the *Clostridium perfringens* iota toxin Ia fragment with actin [24,25]. The Ia-actin interface involves 32 residues from each protein partner and covers 829–895 Å^2^, or about 5% of the surface area of each protein. Furthermore, the interface involves five loops on the Ia fragment, only one of which has amino-acid homology in CTA1 (the ARTT loop), which include residues targeted in this study. Because actin and Gsα are not homologous, we cannot yet compare the relative contributions of amino acids at specific positions (other than the acceptor arginine residue) to the interactions of these substrates with their cognate ADP-ribosylating toxins.

We have shown here, and others have shown [30,31,32], that both *in vitro* and *in vivo* Gsα alone is a poor substrate for ADP-ribosylation, even in the presence of GTP-bound ARFs. Unlike the case for pertussis toxin, where peptides encompassing the last 10–20 amino acids of PT-sensitive alpha subunits (surrounding the target cysteine in Giα) are equally as effective substrates for PT-catalyzed ADP-ribosylation as is native Giα [33], CTA1 likely requires a very particular ternary complex that includes Gsα to enable ADP-ribosylation. Recent crystal structures of G protein complexes have given insight into why this is so. Early work with Gt (transducin) [18] and Gsα [34] produced data that were consistent with the natural substrate for cholera toxin being the activated GPCR-bound, nucleotide-free form of Gsα. Gsα in the GDP bound (inactive) form has a high affinity for Gβγ, and they form a stable heterotrimeric complex. *In vivo*, this complex is tightly bound to membrane-anchored quiescent GPCRs. Upon stimulation by receipt of an activation signal, the activated receptor induces a conformational change that results in opening of the interdomain interface in the G protein heterotrimer with release of GDP, generating a nucleotide-free Gα(0) as part of the ternary complex of activated GPCR-G protein heterotrimer [35]. The structure of this activated complex has recently been determined [36,37] and reveals a remarkable conformational change in the nucelotide-free Gsα upon release of GDP, compared to its structure in the GPCR-coupled GDP-bound heterotrimer [38] or Giα in the isolated heterotrimer [39]. This complex is short-lived, and the Gsα(0) rapidly binds GTP. Gsα(GTP) has dramatically reduced affinity for Gβγ, and it quickly dissociates from the ternary complex and proceeds to activate downstream effectors, one of which in the case of Gsα is adenylate cyclase.

Our *in vitro* system for ADP-ribosylation of Gsα does not include a membrane-anchored GPCR or the upstream components required for its activation. Interestingly, it has been shown that ARF1 can also interact with Gsα and that the amino-terminus of ARF1 is required for CT-catalyzed ADP-ribosylation of Gsα [40], but not the small artificial substrate agmatine [40]. The presence of Gβγ and the other components in our system must be sufficient (but not necessarily optimal) to generate a form of Gsα that is ADP-ribosylated by CTA1. The availability of these new structures of the activated complexes should facilitate future studies on the interactions of CTA1 and Gsα.

## 3. Experimental Section

### 3.1. Generation of CT Variants by Site Directed Mutagenesis

Alanine substitution mutations in the CTA1 coding region of a clone encoding an arabinose-inducible native CT operon (pARCT5) [22,41] were made by QuickChange mutagenesis using the manufacturer’s protocol (Stratagene) (Table 2). Novel restriction sites were co-introduced along with the alanine substitution to enable tracking of changes. Double and triple substitutions were made by subcloning if suitable intervening restriction sites were available or alternatively by a second or third round of QuickChange mutagenesis. Each mutation was confirmed by DNA sequencing of the PCR amplified CTA1 coding region from the mutant plasmid clone.

### 3.2. Protein Production and Purification

Wild type CT holoxin and variants with alanine substitutions in the CTA1 domain were purified from extracts of cells from 400 mL of induced cultures. 50 mL overnight cultures in TB [42] grown at 30 °C were diluted into 400 mL of pre-warmed broth and grown to an A_600nm_ of 3.5 and induced with 0.2% l-arabinose for three hours. Induced cells were collected by centrifugation and resuspended in 1/20th volume of Talon buffer (50 mM Na phosphate, pH 8.0, 0.3 M NaCl) and extracted with 1% Elugent (Calbiochem) detergent. DNA was sheared by brief sonication, cell debris was removed by pelleting at 10,000× rpm at 4 °C, and the resulting extracts were purified by Talon affinity chromatography using the manufacturer’s protocol, except that purified toxins were eluted with only 50 mM imidazole. Holotoxins were purified further by two rounds of ion-exchange chromatography. Pooled fractions from the Talon elution were dialyzed into 50 mM Tris pH 8.0 (buffer A) and applied to an HS20 cation exchange column to remove free CTB penatamers. Holotoxin eluted in the flow-through, and the column was then washed with 5 column volumes (CV) of buffer A. Free CTB pentamers were eluted with a 10 CV 0%–100% gradient of buffer A to buffer B (buffer A with 1 M NaCl). The holotoxin-containing flow-through was applied directly to an HQ10 anion exchange column (to which holotoxin bound), and the column was subsequently washed with 5 CV of buffer A after which holotoxin was eluted with a 10 CV 0%–100% gradient of buffer A to buffer B. Fractions were analyzed by SDS-polyacrylamide gel electrophoresis and peak holotoxin fractions were pooled and dialyzed into buffer A for storage.

**Table 2:** Oligonucleotides used in this study.

Name	DNA Sequence (forward Primer only, Changed Bases in Italics)	Notes and Novel Restriction Site Introduced (Underlined in Sequence)
CTAXF	CGGGCAGATTCTAGACCTCC	amplifies CTA1 for cloning with XbaI
CTACR	CTCATCGATGATCTTGGAGCATTCCCACA	amplifies CTA1 for cloning with ClaI
R25AF	GGTCTTATGCCA*GCT*GGACAGAGTGAG	AluI
T50AF	AAGAGGAACTCAG*G*C*C*GGATTTGTTAGG	HaeIII
F52AF	ACTCAGACGGGA*GC*TGTTAGGCACGATG	AluI
H55AF	GGATTTGTTAGG*GC*CGATGATGGATATG	HaeIII
R67AF	CTCAATTAGTTTG*GCC*AGTGCCCACTTAG	MscI, EaeI(YGGCCR), HaeIII
L71AF	GTTTGAGAAGTGCCCAC*GCG*GTGGGTCAAAC	BstUI
T75AF	CTTAGTGGGTCAA*G*CTATATTGTCTGGTC	AluI
I76AF	GTGGGTCAAACT*GC*ATTGTCTGGTCATTC	HpyCH4V
S78AF	CAAACTATATTG*G*C*C*GGTCATTCTAC	HaeIII
H107AF	GGCATACAGTCCT*GCA*CCAGATGAACAAG	HpyCH4V
D109AF	CATACAGTCCTCATCCAG*C*TGAACAAGAAGTTTCTGC	PvuII, MspA1I (CMGCKG)
E110AF	CCTCATCCAGATG*CG*CAAGAAGTTTCTGC	*FspI*, HhaI (GCGC)

Recombinant ARF6 was made from cytoplasmic extracts of IPTG-induced *E. coli* BL21(DE3) [pT7arf6] [11]. Cells were lysed by four cycles of freeze-thaw in TBS with 0.1% Triton-X100 and lysate viscosity was reduced by brief sonication or treatment with 10 μg/mL DNase I. Extracts were diluted 1:2 with 10 mM Tris-EDTA and applied to a DEAE-sepharose column equilibrated with 20 mM Tris pH 7.4, 50 mM NaCl. The majority of cellular proteins bound to the column, but rARF6 eluted in the flow-through and was then purified further over a second DEAE-sepharose column.

6-his-tagged-Gsα was made from soluble extracts of IPTG-induced cultures of *E. coli* BL21(DE3)[pUBS520, pQE60-his6Gsα] as described by Yan and Tang [43], purified by Talon affinity and Q-sepharose ion exchange chromatography, eluted with 0.1 M–0.5 M NaCl gradient in 20 mM Tris pH 8.0, 5 mM β-mercapto-ethanol, 0.1 mM PMSF, 1 mM MgCl_2_. Peak fractions were pooled, concentrated and buffer exchanged with 10,000 Da cutoff Amicon filters into 50 mM Tris pH 8.0, 1 mM EDTA, 1 mM DTT and 10% *v/v* glycerol, aliquoted and stored at −70 °C. Functionality was tested in a GTP-binding assay using fluorescence of BODIPY-GTP [44].

Recombinant Gβ1γ2 was purified from Sf9 cells infected with Bacmid clones expressing his6-Giα1(G203A), Gβ1 and Gγ2. Genes for the G-proteins were cloned into pFastBac1 and transposed into the bacmid backbone in DH10Bac cells with the Bac-to-Bac system from Invitrogen. Recombinant bacmids were confirmed by DNA sequencing, and transferred to the UCD tissue culture/Mab core facility to produce heterotrimeric G-protein complex by co-infection of Sf9 cells. The complex was purified from Sf9 cell membranes by Talon affinity chromatography, and Gβ1γ2 was eluted and purified from the complex as described [45].

### 3.3. Enzymatic Assays

Wild type and variant CT holotoxins were first activated by limited trypsin digestion. Samples of holotoxin were treated with 1/10^th^ *w/w* bovine trypsin and incubated at 37 °C for 30 min; trypsin was inactivated with the addition of 2× *w/w* soybean trypsin inhibitor; and the resulting samples were stable at 4 °C. Enzymatic activity of variant holotoxins was measured quantitatively as previously described [46] using the small artificial substrate DEABAG at 2 mM, which is near its solubility limit [22]. All results were initially expressed as background-subtracted fluorescence units ± SEM/assay (9988 ± 50 for wt); and the results for variant toxins were then normalized to values set to 1.0 for the results with wt CT. Reactions with Gsα as substrate were done in 60 µL volumes of assay buffer (50 mM potassium PO_4_ pH 7.5, 1 mM MgCl_2_, 3 mM DTT, 20 mM thymidine, 100 µM GTP, 10 µM NAD) [47,48] with 2 µg of activated wt or variant CT, 2 µM rhis6Gsα, 0.5 µg rGβγ, 2 µg rARF6 and 0.5–1 µL ^32^P-NAD (5 µCi; 800 Ci/mmol; 5 mCi/mL). Reactions were incubated for 1 h at 30 °C, stopped by precipitation with TCA to also remove excess label, and washed with cold acetone. Pellets were resuspended in 30 µL PBS and analyzed by SDS-PAGE. ^32^P-labeled Gsα at approximately 45 kDa was quantitated on a Biorad Phosphorimager, expressed as background-subtracted counts/mm^2^. Signals for equal amounts of individual CT variants was then normalized to wt = 1.0.

### 3.4. In Vivo Toxicity Assays on Mouse Y1 Adrenal Cells

Y1 cells (ATCC CCL-79) were cultured in RPMI-1640 medium with 10% fetal calf serum in a humidified 5% CO_2_ atmosphere. Cells were grown to semi-confluence in 96-well plates. Intracellular cAMP levels were quantitated in duplicate from acidic ethanol extracts of activated toxin-treated cells (10 ng/mL, 100 µL/well for 2 h at 37 °C) using a Cayman Chemical cAMP EIA kit (581001) as described by the manufacturer; values were then normalized to wt (440 ± 13 SEM pmol/well; no toxin (basal signal) 40 ± 26 SEM pmol/well).

## 4. Conclusions

By rational design, site-directed alanine mutagenesis, and phenotypic characterization of wt and mutant forms of CT, we identified several individual amino acid residues in CTA1 (H55, R67, L71, S78, and D109) that are likely involved in recognizing and binding the native substrate Gsα. Single alanine substitutions for any of these surface-exposed residues had little or no effect on the ADP ribosylating activity of CTA1 for the small acceptor substrate DEABAG (which likely interacts within the active site cleft of CTA1), but they caused decreases in the ADP-ribosylating activity of CTA1 for the native acceptor substrate Gsα (a protein that likely binds to CTA1 by surface interactions during the process of ADP ribosylation of the target arginine-201 residue in Gsα). In addition, CT variants with a single R25A, F52A or T50A substitution had moderately decreased activity with DEABAG but severely decreased activity with Gsα, indicating that they likely participate both in catalytic activity and recognition of Gsα.
